# Towards Post-Pandemic Sustainable and Ethical Food Systems

**DOI:** 10.1007/s41055-020-00084-3

**Published:** 2021-01-23

**Authors:** Matthias Kaiser, Stephen Goldson, Tatjana Buklijas, Peter Gluckman, Kristiann Allen, Anne Bardsley, Mimi E. Lam

**Affiliations:** 1 Centre for the Study of the Sciences and Humanities, University of Bergen, Bergen, Norway; 2 Koi Tū Centre for Informed Futures, University of Auckland, Auckland, New Zealand

**Keywords:** Food systems, Food ethics, Sustainable food, Transdisciplinary research, Farm-to-fork

## Abstract

The current global COVID-19 pandemic has led to a deep and multidimensional crisis across all sectors of society. As countries contemplate their mobility and social-distancing policy restrictions, we have a unique opportunity to re-imagine the deliberative frameworks and value priorities in our food systems. Pre-pandemic food systems at global, national, regional and local scales already needed revision to chart a common vision for sustainable and ethical food futures. Re-orientation is also needed by the relevant sciences, traditionally siloed in their disciplines and without adequate attention paid to how the food system problem is variously framed by diverse stakeholders according to their values. From the transdisciplinary perspective of food ethics, we argue that a post-pandemic scheme focused on bottom-up, regional, cross-sectoral and non-partisan deliberation may provide the re-orientation and benchmarks needed for not only more sustainable, but also more ethical food futures.

## The Disruption

The world is experiencing the greatest collective disruption since World War II. The novel coronavirus SARS-CoV2, causing the disease COVID-19, has disrupted production and mobility of people globally. New concepts, such as social distancing, permeate the public consciousness. Science, notably through virology, epidemiology and vaccinology, is called upon to solve the acute health dimensions of the current crisis. The social sciences, and particularly the behavioural sciences (van Bavel et al. 2020; Lam in press), have been used more sparingly to set policy in most countries (Lavazza and Farina 2020; Leslie et al. 2020), while in Germany, philosophers and other humanities scholars also have been recruited (Matthews 2020). Countries are preparing for long-term economic challenges, even worse than the financial crisis of 2008–9 and the Great Depression of the 1930s. It is projected that both State and business debts will increase considerably, as will protectionism, while global travel (Hall et al. 2020) and trade will decline markedly, accompanied by shorter supply chains (Bonadio et al. 2020) and greater volatility in the pricing of stocks, property and currencies (Mirza et al. 2020; Corbet et al. 2020). Consequently, globalization and free trade are threatened. The major trends shaping the geopolitical landscape prior to the pandemic are being exacerbated, namely: the USA is withdrawing from global leadership and support of the multilateral rules-based order^1^; the European Union is struggling internally to re-define itself; China is focused on extending its influence and improving its reputation, and Russia is seeking to regain its historical position as a major power. Meanwhile, the global South is awash in the pandemic and other humanitarian crises, with food insecurity looming large (Tisdall 2020; Mishra and Rampal 2020).

One of the few good things arising from this pandemic has been a sharper focus on those areas – and indeed, values – of life that matter most for people. Through the pandemic, underlying value dilemmas have come to the fore, instigating critiques of some sectors of society and revealing the dire need to resolve such dilemmas to overcome global challenges. One such value-laden area where we all want stability and security is the global food system.

In this paper, we suggest that the current global crisis provides an opportunity to engage in broad discussions of how to re-configure our global food system, which encompasses both terrestrial and aquatic food production. Rather than defining a single goal or solution, or even a single set of global solutions, we argue for more nuanced, yet structured deliberation to re-configure the overarching framework and processes that underpin the global food system. We argue that coherent strategies for sustainable and ethical developments in the future food system need greater grassroots involvement, cross-sectoral engagement, and transdisciplinary expert-advice. Fundamentally, more decisions should be adjusted to reflect local and regional conditions rather than global benchmarks, and be explicitly tied to people’s value-landscapes or value priorities.

The re-orientation we propose of food systems mirrors some of the essential ingredients captured in ethical soundness (Kaiser et al. 2007), deliberative democracy (Elstub 2010), good governance (van Doeveren 2011) and ethical governance (Lam et al. 2019), namely: legitimacy, accountability, participation, fairness, equity, transparency, and value pluralism. But expanding upon these, we aim at a comprehensive food ethics by stressing the inherent value plurality specifically among food actors and the need to make these value orientations explicit within a democratic deliberative process guided by procedural ethics. The explicit inclusion of scientific, but also societal uncertainties inherent in value plurality is crucial for this endeavour. While such an ethical framework is useful generally in many areas of life (e.g., Millar et al. 2007), it is of particular importance for the global food sector, given the high stakes that lie at its confluence of global food security, health, environment and trade.

A crisis, such as the world is now facing, puts elemental values in focus again, and since most socio-economic and socio-political routines are disrupted, opportunities for reforming the food system are emerging from the disruption. A lesson from history and psychology is that a crisis can induce behavioural changes in people. Specifically, the current crisis is revealing, among other things, consumers’ attitudes and perceptions about the food system. At the onset of the COVID-19 crisis, consumers’ fears of food shortages led to panic-buying and hoarding behaviours, fears which since have proven to be unwarranted. For most people, obtaining enough food has not been a problem, notwithstanding increases in those dependent on food banks and soup kitchens during the pandemic. From media accounts, it appears that the food supplies in most countries were able to meet even such heightened demands, with stored food inventories typically ranging from three to four months. The price spikes and increased hunger of the 2007–8 rice crisis have not been repeated in the pandemic, though recent export controls of rice from Vietnam did increase prices sharply.^2^

The pandemic has exposed several sustainability and ethical challenges to the food system. For example, many farms and production facilities are highly dependent on processing or slaughtering capacities, industrial services that include seed, feed and fertilizer availability, and cheap, seasonal, mostly immigrant labour. Disruptions to any of these could interrupt food availability and flows. Similarly, with most planes grounded and many ships docked, the viability of global food transport is threatened by an uncertain post-pandemic future. Food is a highly traded global commodity, but this global trade can be compromised by heightened unpredictability and instability of financial markets due to low and volatile oil prices and rising unemployment. Furthermore, consumer dining patterns shifted to home-cooking and take-outs during the pandemic while restaurants and schools were closed, but it remains to be seen if any lasting trends in consumer food preferences will emerge in the post-pandemic era.

## An Ethical Lens: Plurality of Food Values and the Need for Deliberation

The current crisis brings critically to the fore underlying food value dilemmas that need to be resolved at multiple scales. This requires a deliberative framework grounded in procedural ethics for re-envisioning and re-structuring the food system. Foremost, deliberation can make explicit the plurality of values of diverse food actors and offer mechanisms to facilitate open, respectful dialogue. The aim of the deliberation would be to prioritize together potentially divergent values and to resolve value conflicts that may emerge. Coherent plans of action can be articulated, grounded in the best available science with agreed upon benchmarks capable of measuring and if needed, recalibrating progress towards identified common goals.

To avoid potential misunderstandings, we stress that our focus on ethical deliberation does not imply a universal normative ethical theory, or even a unique definitive ethical outcome. Rather, we view this as the basis of a general ethical framework in which food actors seek to design a common strategy for how to respect and secure the values endorsed by all actors. Societal decision-making with accountability would then aim to live up to this context of social norms and values, and to avoid disapproval from not communicating to relevant others. The art of good and ethical decision-making thus consists in finding reasons that others take as convincing: “decisions under the accountability model are more likely to follow a reason-based rather than a rule-based mode” (Keren and de Bruin 2003, p. 358). These ethical qualities are captured by the deliberative processes fulfilling some criteria of “ethical soundness” (Kaiser et al. 2007), notably the explicit inclusion of the value landscapes of societal actors and the rational assessment of options given these values and commonly agreed norms.

We argue that food actors from the government, market, civil society, and law should participate in the decision-making and governance of food systems (Lam and Pitcher 2012) to reflect broad societal values. Generally, market institutions and values have dictated food availability and choices, with eco-labelling schemes emerging to capture non-market consumer values, such as sustainable seafood and fair trade (Lam 2016; Haugen et al. 2017). Increasingly, however, even such market-based tools are seen to be problematic, with third-party assessments often favouring industrial-scale enterprises (e.g., Jacquet et al. 2010). Thus, non-market food actors, such as inter-governmental, governmental, and non-governmental organizations, as well as professional societies and individual scientists need to be aware of the plurality of values influencing food decisions and to be open to deliberative processes that promote ethical food governance. For example, the Food and Agriculture Organization of the United Nations (FAO) and the International Council for the Exploration of the Sea (ICES) are two intergovernmental organizations that regularly convene meetings of scientists and policymakers with stakeholder representation from industry, but less so from civil society. Governmental organizations responsible for regulating fisheries (e.g., Department of Fisheries and Oceans Canada and the National Oceanographic and Atmospheric Administration in the USA) routinely host participatory consultations with broad stakeholder interest groups, but the process is often top-down. NGOs, such as World Wide Fund for Nature, attempt to influence global policies to reflect conservationist values through stakeholder processes, but remain vulnerable to influences from powerful actors (Havice and Iles 2015). Professional organizations, such as the European Society for Agricultural and Food Ethics (EurSAFE; www.eursafe.org), and scientific teams can highlight ethical considerations in the local management and governance of natural resources, such as in fisheries (Lam et al. 2019), or can address global food systems to promote plant-based diets, but lack ethical nuance, such as in the EAT-*Lancet* report (Willett et al. 2019).

An ethical deliberative framework for food systems would include these key elements:

1. Invite early all relevant food actors at national and regional levels. Conversations should be cross-sectoral, cross-cultural, and open to plural worldviews and values with ethical deliberations of how to re-prioritize and re-structure the food system.
2. Start with framing the problem as it appears in each constituency / region to reveal implicit values. Global benchmarks should be noted, but contextualized to the specific regions. Seek tailored options that work for the specific target systems.
3. Be politically non-partisan to seek common understanding among the actors and transparency for diverse sectors and publics within society.
4. Invoke the best science, as represented in a transdisciplinary effort shaped by refocused scientific education and research that is fit for (societal) purpose.
5. Adopt a post-normal science approach^3^ to the food system, as the facts are uncertain, values are disputed, the stakes are high, and decisions are urgent.

The last three essential elements in the deliberative process need some elucidation. First, the notion of being politically non-partisan implies that the process does not adopt the program of any political party or governmental policies. It should remain neutral and open for future food scenarios as envisioned by various actors seeking to influence policy, not presuppose political strategies. EurSAFE, for example, inscribes non-partisanship in its mandate. Second, the notion of transdisciplinary expertise is wider than mere interdisciplinarity, and focuses on a genuine dialogue among experts and diverse stakeholders and citizens. This implies, inter alia, that the framing of the problem is not limited to disciplinary or cultural framings (cf. Lam et al. 2019; Kaiser et al. 2020; OECD 2020). Third, a post-normal science approach (Funtowicz and Ravetz 1990, 1993) implies not only awareness of the need for adequate quality in societal decision-making, but also reflexivity of the inherent uncertainties and contestation of values and facts. It widens the scope of what constitutes relevant expertise to include society in science for policy.

The current global food value chain is driven not by ethical deliberation, but often implicit competing values and power dynamics, where influential global actors favour profit motives over non-market values, such as food equity, sovereignty and justice. We hypothesize that the introduction of an ethical deliberative framework into the food system would help to prevent distributional issues, such as food inequity, which the pandemic has exacerbated. In the subsequent sections, we first highlight the prevailing pre-COVID global food system. Next, we show how the current pandemic has exacerbated inequities. We conclude by proposing, as an optimistic ‘new normal’ for post-COVID, ethical deliberation that recognizes a plurality of food values. Ethical deliberation within the global food system would constitute both a prelude to decision-support tools and a cornerstone to the ethical governance needed to resolve inherent value conflicts in food policy and governance.

## The Pre-Pandemic Situation

Long before the pandemic amplified issues, numerous reports and global assessments had already concluded that the global food system per se is not sustainable and in need of radical change (FAO 2018b; Costello et al. 2019; SAPEA 2017, 2020; Willett et al. 2019; Odegard and Van der Voet 2014). The recent SAPEA report ( 2020) stated boldly: “‘business as usual’ is no longer a viable option and radical change is required” (p. 21; also FAO, F 2018a). The EAT-*Lancet* report (Willett et al. 2019) called for a re-assembly of our diets along globally defined goals. An earlier SAPEA report ( 2017) introduced the concept of “smart eating” as the answer to resource and health responsibility (cf. Scherer and Holm 2020). So-called political or ethical consumerism in industrialised countries (Micheletti 2006), driven forward by consumers and NGOs, and amplified through the choice editors of supermarket chains, is putting animal welfare, sustainability, environmental degradation, and food waste on food policy agendas, resulting in emerging niche markets and new certification labels.^4^

Holistic thinking has entered food policy debates through concepts such as the One Health approach/movement, which links human, animal, and environmental health (Huth et al. 2019; van Herten and Meijboom 2019), and Food Sovereignty, which aims at culturally adapted control of mechanisms and distribution of food by those who produce, distribute and consume foods (Rosset 2008). Countries have established effective institutions to guard food safety issues (e.g., the Food and Drug Administration in the USA and the European Food Safety Agency in Europe), which have resulted in improvements to food safety, though various foodborne diseases still represent significant problems even in industrialised nations (EFSA and ECDC 2018). Meanwhile, trade globalization (and climate change) have introduced new pest and disease species into ecosystems without resident natural enemies and this is adversely impacting food production and environmental quality (e.g., Cock et al. 2012; Ferguson et al. 2018). Despite integrated pest management systems, which limit the use of pesticides through biological control and breeding for plant resistance (e.g., Goldson et al. 2020), food systems remain vulnerable to further disruption through the arrival of yet more transported pest species.

**Fig. 1:**
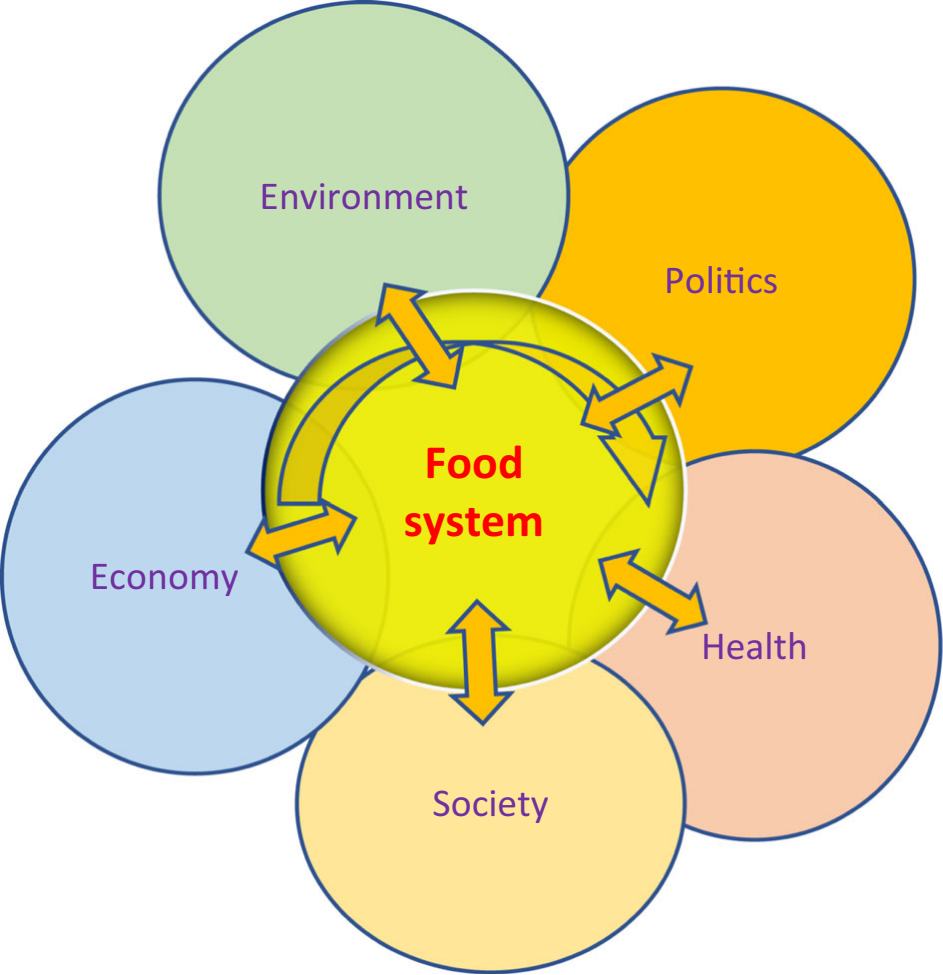
Circular map of the food system, with flows connecting the food chain with the environment, economy, society, health, and politics, inspired by and simplified from Parsons et al. 2019. The point of the figure is to show that at each point of the food chain there is interaction with each of the five surrounding systems, which also interact with each other

What once was dogma is now seen by many as the root of the problem. The (in)famous aphorism of Earl Butz “Get Big or Get Out!” characterized the conventional paradigm in food systems of intensive farming, monocultures, and mass consumption along linear value chains linking food production, processing, distribution, retail trade, and consumption. Recent efforts have examined the ecological sustainability and ethical issues that arise among social actors within complexly interacting global food chains, such as seafood value chains, and their implications for food governance (Lam 2016). The debate around GM-food has highlighted the complexity within our food system and the confounding, but essential role of consumer perceptions and marketing in shaping food value chains. Many experts now agree that assumed linearity is inadequate to capture the complexities of the food system, and that the food system is more appropriately portrayed as a potentially circular and interlocking system interacting with a variety of other social and natural systems (cf. SAPEA 2020; see Fig. 1). More complex, adaptive and circular systems of food chains are recognized, overlapping with the environment, economy, society, health and politics, themselves complex systems which also interact with each other. For example, the food system can be viewed as the nexus or interlocking of systems of food, water and energy. It is this generalized, complexly interacting framework of the “food system” to which we refer when using this term, rather than any specifications by nation, region, or products, which necessarily would introduce contextual variance. While we recognize that the governance of any particular food system would need to account for this contextual specificity, we focus here on establishing the ethical principles of a generalized framework for food systems.

Food experts in agriculture, livestock, aquaculture and fisheries are moving away from conventional linear thinking to recognize food systems as complex adaptive systems.^5^ Policy principles like the Precautionary Principle^6^ often are invoked and interpreted to deal with this complexity, but are rarely translated into practice and indeed have different meanings to different individuals. Since complex systems typically display significant inherent uncertainty (not only technical, but also methodological, epistemological, and societal uncertainty; Maxim and van der Sluijs 2011) and unpredictability due to non-linearity and emergence, the Precautionary Principle offers an ethics-based incentive to deal with value trade-offs and considerations geared to reduce or eliminate the potentially harmful effects of the system. For instance, climate change has intensified food debates, with many advocating the benefits of reducing meat consumption to lower greenhouse gas emissions, but translational efforts to introduce new regimes of sustainable agricultural practice have hit economic barriers.^7^ The FAO ( 2018b) is embracing the message that humanity needs to increase food yields and volumes to feed more people: “producing more will be unavoidable, and the way forward is doing so with less” (FAO 2018a, p.14). Yet, critics have highlighted political bias and power games in the data and measures used by ‘neutral’ organizations such as the U.N. (see Pogge 2016). We agree that global population growth may necessitate increases in food production volume and reductions in food waste can help feed the world. However, new technology, such as in production or waste treatment, alone cannot solve the food gaps that are emerging. Human dimensions, including cultural factors, and integration across diverse value-systems will prove decisive for robust future-oriented food strategies.

The prevailing narrow conception of values in the global food system has been criticized (e.g., Thompson 2010, 2016; Korthals 2004; Lam 2016, Lam 2019a, 2019b). We build on these critiques to highlight the plurality of values that need to be considered and prioritized in ethical food systems. Given its importance to many national economies, the most salient food value is typically the financial value determined by the trade of food products in commodity markets. But non-market values, such as societal, ecological and ethical values, are also associated with the food sector. One such value is the opportunity for meaningful employment and income for diverse food workers, encompassing men and women, rural and urban dwellers, and those with low and high education. Societal values related to human wellbeing*,* like food safety and food security, seek to eradicate undernourishment and obesity, the twin extremes of malnutrition. Another value is the preservation and maintenance of cultural landscapes and lifestyles in a country to retain some of the integrity of the natural history. Significant ecological value underpins the entire food system, as revealed when, for example, monocultural production systems destroy agricultural soil structure; pesticides adversely impact biodiversity leading to pollinator decline; and chemical pollution and eutrophication from open-pen aquaculture create anerobic dead zones. Problematically, single-species fisheries stock assessments ignore the often complex ecological relationships in marine food webs, such as that link predators with prey and other indirect interactions that can induce cascade effects and alter primary production.

Ethical values are implicitly embedded in institutional approaches to the food value chain. Animal and human welfare are obvious ethical issues. The working conditions within food supply chains have been highlighted by highly publicized cases of forced labour in fisheries (Chantavanich et al. 2016; Simmons and Stringer 2014), as well as viral outbreaks and labour issues among dependent, low-paid, often migrant workers in meat production and processing facilities during the current pandemic. Pervasive in these global (economic) food systems is a lack of respect for the integrity of nature and the dignity of workers, often marginalized or silenced voices in today’s identity politics (Fukuyama 2018; Lam in press). Similarly, indigenous food traditions and cultural values often are disrespected, as they may conflict with prevailing worldviews or underlying western notions of property (e.g., Lam et al. 2019; Maxwell 2019).

In sum, many aspects of the modern food system represent ethical transgressions by ignoring pervasive, yet often implicit values of food actors lacking political power. Our ethical accounting of the food system calls for a radical change towards the explicit inclusion and deliberation of this plurality of values to reconcile inherent value conflicts among diverse food actors in the global food system. Systemic change is legitimate and urgently needed to reflect the full suite of food values implicit in the three basic pillars of sustainability, namely economic, social and environmental. Through identification and explication of these values and deliberation to reconcile inherent value conflicts, food systems can become not only more sustainable, but also more ethically governed.

## Shortcomings of Science and its Administration in the Food Sector

Here, we criticize the current application of science in the food sector, while recognising its vital importance for further development. We perceive the core issue to be that science funders fail generally to appreciate the values inherent in problem-framings and the scope of science needed to address societal problems (cf. Saltelli et al. 2020a). Much of science funding originates from political-economic actors who then can influence the direction of the science being done. Irrespective of the funding sources, science often operates within constraints and objectives set by non-scientific, but elite politicians, bureaucracies, and lobby groups who may even deem what constitutes acceptable data in science for policy.^8^ For instance, the FAO claims to provide authoritative overview of the global state of food production, while nation states contribute data as they pursue strategic objectives focused on production volume, value and exports. Similarly, NGOs, as civil society organizations, are social institutions defined by their values (Turner Jonathan 1997).^9^ They thus pursue selective value-based objectives, as reflected in their reports, and can be blind to data and findings that run counter to their ideologies. NGOs claim to serve the role of societal guardians by funding research and advocating for social or environmental causes that align with the putative values and interests of society (Lam and Pitcher 2012). But NGOs are interest groups with political agendas that may be at variance with the societal good, notably when international NGOs pursue initiatives that promote their own ideologies or the interests of their donors (Tortajada 2016) at the expense of the values, norms or interests of local communities. When scientists align their research with the bias of funders, authoritative bodies, or powerful elites, they gain access to research funding and voice in high-level discussions, but may compromise their ethos of objectivity (Merton 1942). This is a pervasive, yet critically unexamined ethical issue within science.

We also contend that too much of the science of food systems adopts modelling protocols based on neo-classical economics. Many sectoral strategies developed by the industry or governments tend to use growth models that naively extrapolate from earlier developments. This may be due to a path dependency in science that favours certainty (i.e., low-risk science), but offers little real progress, yet unfortunately characterizes many current extrapolations of future trends. This constrained approach is vulnerable to the “Black-swan” problems, as coined by Taleb (2007), i.e., the occurrence of rare, hard-to-predict, but consequential events in complex systems. In general, such simplified modelling frameworks ignore the complexity and uncertainty in data and thus can seldom account for non-linearity in development and sudden changes of state, as often typified by real-world events, such as the current pandemic.

One reason for this blindness to complex food realities is that most scientific research is confined to the inner logics of disciplinary silos, where careers are built and funding allocated based on metrics favouring publication and impact within disciplines. Transdisciplinarity is an emerging concept, which is still too little practiced or valued within the scientific community. It is in societal crises, such as the COVID-19 pandemic, that the merits of transdisciplinarity in science-for-policy are revealed. However, academic education, scholarly rewards, and research funding still operate on the disciplinary logic of post-WWII, pre-pandemic times.

These factors have culminated in domains of public health and nutrition looking to global outlooks, solutions and strategies, effectively “silver bullets” that their researchers and funders argue will provide sustainable and ethical futures across the whole food sector. For example, the EAT-*Lancet* report, authored by a global team of scientists (Willett et al. 2019), claims to provide a science-based solution to feed a world of 10 billion people with a healthy and environmentally responsible diet for all. They develop universal scientific targets for healthy diets and sustainable food production and integrate these into a common framework, “the safe operating space for food systems” (ibid., p. 7). Their approach problematically uses global aggregate indicators (criticized in Giampietro and Saltelli 2014) and models built on faulty assumptions (Saltelli and Funtowicz 2014; Turner and Gardner 2015), while neglecting basic uncertainties in knowledge about nutrition (Archer et al. 2017; Ioannidis 2013) and large spheres of social science research (see SAPEA 2020).

We raise five issues with the EAT-*Lancet* report. Firstly, it only glancingly refers to people and their mores, thus ignoring the social importance and cultural embeddedness of food, and is biased to affluent populations in the Western World, blind to the diverse realities of primary producers and their local, temporal, and situational contexts. Secondly, the report makes exaggerated claims, such as 11 million lives saved annually,^10^ without satisfactory scientific evidence (cf. Brown et al. 2014). Thirdly, it operates with precisely quantified targets of healthy nutritional intake, despite their extreme uncertainties (cf. Schoenfeld and Ioannidis 2013; Brown et al. 2014) and historical shifts (Archer et al. 2017). Fourthly, the report proposes top-down global solutions, which developmental economics has shown to be ineffective in implementing changes in production, technology and management (Cooke and Kothari 2001; Cornwall 2006), ignoring the critical adaptivity and bottom-up dynamics of local and regional stakeholders to develop resilient, functioning food systems. Lastly, it gives the impression of providing instrumental help, i.e., concrete strategies for realizing the goals, while only offering truisms (e.g., “commitment to healthy diets”). Thus, even this most celebrated work on food sector development largely fails in terms of scientific quality (i.e., fitness-for-purpose) and applicability, and hence lacks societal and policy resonance.

Food policy presents us with a ‘wicked problem’, where “problem understanding and problem resolution are concomitant to each other” (Rittel and Webber 1973). That is, the framing of the problem, which depends on one’s values, will bias the proposed solutions. This factor alone should warn the scientific community to remain vigilant regarding their own values, paradigms, and limitations of knowledge. It is imperative to listen to the needs and values of the intended users, as well as to dialogue with experts from other fields. Otherwise, the risk is high of perpetrating a Type III error (Mitroff and Featheringham 1974), colloquially expressed as: “Very good science but, unfortunately, altogether the wrong problem!”

These shortcomings of science-for-policy have been accentuated during the current pandemic, where many scientists enter the public forum with often divergent claims and abstract models based on routine (normal-science) procedures. Science-for-policy operates within ‘post-normal science conditions’ (Funtowicz and Ravetz 1990, 1993; Gluckman 2014), namely: many “facts” are highly uncertain, the relevant values are in dispute, the stakes are high, yet the decisions are urgent. To respond to such conditions requires transdiciplinarity and broadly inclusive dialogues (see Lam et al. 2019; OECD 2020; Kaiser et al. 2020). Traditional disciplinary approaches have been limited by problem-framings that are not necessarily those of the decision-makers or society. Far too often in the current crisis, scientists have appealed to the “authority” of science without reflecting on these points (Saltelli et al. 2020b). This ethical lacuna persists also within most future food policies.

## Pandemic Response in New Zealand: Emerging Cause for Food Optimism

In response to the pandemic, individual countries are re-orienting to develop robust and resilient strategies, also in the food sector. For large countries or federal states, this task may seem insurmountable due to the variety of interests and often the lack of social cohesion. Smaller countries may have an advantage, as they can more easily mobilize broad sectors of society and thus serve as natural laboratories for socio-political change.

In New Zealand, where we were in lockdown while writing this paper,^11^ we are witnessing a social-political and scientific experiment born in the crucible of crisis. The *Koi Tū Centre for Informed Futures* has produced an orientation paper, entitled ‘The future is now’, as a framework to jumpstart strategic discussions of post-pandemic times.^12^ It has initiated broad (online) discussions about major sectors of public life, including the future of the food sector in New Zealand (*Koi* Tū 2020). The *Koi Tū* has mobilized diverse expertise and perspectives from within the food sector, such as agriculture, fisheries, aquaculture, food processing, environmental protection, retail, science, and *Māori* values.

The New Zealand-style of pastoral production, situated at the nexus of precipitation and water management, agriculture, and aquaculture, exemplifies pertinent value considerations and food perspectives. Economically viable sheep and beef farming is often found in steep, hilly, rural land that is not suitable for other production. Conversely, some intensive dairying cannot avoid ecosystem damage when it occurs where the soil structure is fragile and free-draining. Management questions are arising regarding past land-use zoning decisions and some irrigation schemes. Additionally, such pastoral and arable land planning failed to factor in the large shift in rainfall patterns expected over the next 30 years due to climate change.

This confluence of factors and complexity calls for refocused dryland research^13^ and revised food thinking and oversight, initializing some notable outcomes. Firstly, a significant degree of concurrence exists among food actors that major change is needed, in alignment with the SAPEA conclusion that business-as-usual is no longer a viable option. Secondly, New Zealand high-level actors, notably industry leaders, strongly objected to politicising this debate about the future of the food system and have called for a non-partisan platform (namely, the *Koi Tū*) to facilitate the cross-sectoral discussion in a de-centralized and inclusive manner. Thirdly, while these actors were, and still are critical of single-issue mainstream science dealing with the food system, they recognize the need for scientific input that is transdisciplinary. Fourthly, while global analyses can be useful as a rough orientation and political reference point, their use as forward-looking national or regional guides for action to reform the food system is limited. The clear consensus among New Zealand’s food leaders is that a discussion about the national and export-oriented renewed food future is needed now, with scientists who have learned to listen and to dialogue respectfully. Food is seen as too important to be sacrificed in a political melee: political parties and governmental authorities can be participants, but should not be the convenors in food deliberations.

What precipitated deliberation towards a radical re-orientation of the future food system in New Zealand? With a government that responded proactively to the pandemic and with citizens trustful of policy interventions, the island nation was able to prevent its society and economy from being crippled by COVID-19. Nonetheless, the pandemic exposed the country’s vulnerability as a food exporter within the global market. Consequently, New Zealand’s leading food actors responded to the crisis by convening discussions facilitated by the new *Koi Tū* Centre led by one of us (PG), chief scientific advisor to three former New Zealand Prime Ministers. This embodiment in the *Koi Tū* Centre of scientific expertise, socio-political capital, and institutional capacity, combined with a public health crisis and industry desire to avert an economic crisis in the food sector, were galvanizing forces to shift diverse food actors from their prevailing mindsets to collaborate on defining a ‘new normal.’

## The Post-Pandemic Challenges in the Food Sector

While still in the midst of the pandemic, the ‘new normal’ is yet to be born and thus cannot be predicted.^14^ However, to prepare for an uncertain future, we need to make best guesses based on the present. In principle, three possible future food scenarios can be envisioned for post-pandemic times:

1. The ‘new normal’ will revert back to the ‘old normal.’
2. The ‘new normal’ will accelerate existing trends and realize predicted futures.
3. The ‘new normal’ will rupture from the past and radically reset most of society.

Scenario 1 is assumedly the alternative for which many societal actors hope, but also is the most unlikely, given the circumstances of the pandemic. Whether Scenario 2 or Scenario 3 is realized will depend largely on the manifold mid-term economic repercussions of the pandemic. Preparing for the post-pandemic future in the food sector thus will involve strategies that allow adaptation to either possibility. But the current mid-pandemic situation already provides some guidance as to how to meet this strategic challenge.

Here, we list some optimistic trends that we believe can guide the food sector towards a new post-pandemic normal comprised of more sustainable and ethical food systems. Obviously, the trends we emphasize are both subjective impressions and fragmented, but several of these orientations were highlighted in the New Zealand discussions (*Koi* Tū 2020) or are emergent themes percolating in our own research in science-for-policy. Most notable is the strong evidentiary link emerging between sustainability and resilience of food systems. Encouragingly, food systems are being recognized as integrated systems that represent both market and non-market (e.g., societal, ecological, and ethical) values, that is, a plurality of values among diverse food actors that must be reconciled. Striving to achieve food systems that are ecologically viable, socio-economically feasible and societally desirable (Lam et al. 2019) intermingles sustainability with ethics, which we believe is paramount to re-orient food systems to a new post-pandemic normal that does not harm humanity or the environment.

Increasingly, resilience thinking is replacing a simple focus on efficiency in economic, industrial, and notably food systems. Resilience is defined as the capacity of a system to continue providing a function over time despite (unpredictable) disturbances, which is essential for sustainability (Tendall et al. 2015). As a recent article observes, efficiency “may be neither robust nor just. Efficiency can bring fragility” (Rana Foroohar, May 4, 2020).^15^ Post-pandemic supply chains in many sectors will need to be re-evaluated: in the food sector, provision of material to farms and the food-processing industries will likely be re-configured.^16^ All food chains will need delineation, prioritization, and proportionate allocation of supplies to add future resilience to the sector. Governments may mobilize, in accordance with citizens’ expectations, a shift to regional or national food production and processing to meet basic domestic needs. Domestic protection from unreliable global market flows may not be possible, however, due to existing infrastructure and associated sunk costs. Regardless, some deglobalization of food trade is inevitable. Many nations are now sensitized to securing their food resilience in the event of future crises by reviving protectionism and becoming more self-sufficient. But all this is still open for debate. Some argue that climate change and public health are the perfect engines for a revised understanding of globalisation (e.g., Rodrik 2020). However, the degree of free trade envisaged by international bodies, such as the WTO, APEC and OECD, may yet be curtailed by national responses to the pandemic. Even before the pandemic, reform was looming: “Trade liberalisation’s compatibility with sustainability goals remains disputed” (SAPEA 2020 p. 26). Contrary to common rhetoric, the winners could be the poor countries of the global South “by inserting an increasing returns sector of a certain minimum size and diversity into the national labour market” (Reinert 2007, p. 287), while trading with other comparatively poor countries (ibid. p. 280).

Pre-pandemic industry practices and innovations were driven largely by market values, but the pandemic has focused attention on the non-market values and indirect effects of food systems, particularly in terms of health. Post-pandemic consumer demands for healthier food production may rise significantly, while non-compliance to expectations of food-trading nations could trigger more political and/or economic sanctions. For instance, the history of zoonosis^17^ reveals that production modes of domestic (and wild) animals can affect human morbidity and mortality, which warrants stricter food regulations. Environmental health increasingly is being fused with climate change, which may stimulate low greenhouse gas emissions to become a norm in agriculture and fisheries. In societies with indigenous populations, this may have re-distributive food consequences vis-à-vis cultural rights and opportunities. Culturally anchored foodways may be strengthened by decreasing globalized processed or fast food (Banik 2019). Back-to-basics and simplified food habits, such as more home-cooking and less restaurant-dining, could become the norm even in non-pandemic times. While this will not obviate the need for industrial food production and large-scale agriculture, it may diversify food production and consumption to enable niche markets. Knock-on effects may be greater recognition of the unpaid labour of women in the home and greater value to the post-harvest work often performed by women in small fish food chains.

Rarely factored into food regulations and policies, the ethical implications of food systems are slowly being recognized. The ethical concepts of smart food and smart eating are gaining traction in several communities. Food waste reduction is being prioritized,^18^ with food waste prevention initiatives emerging in Europe during the pandemic.^19^ Plastic packaging of food, while welcome in the pandemic, could be reduced in the future and replaced by other media as public awareness rises of the harmful impacts on marine life of microplastics in the oceans. Cultivating algae and filter-feeding aquatic organisms, which do not rely on fish feed, is being promoted over finfish and shrimp farming (SAPEA 2017; Kaiser 2012; Lam 2019b). More environmentally friendly aquaculture systems, such as closed recirculation systems (Meisch and Stark 2019), off-shore production, and integrated multi-trophic aquaculture with smaller shrimp and mussel production ponds, are being investigated, given their benefits to local populations. They are not yet economically viable though, as the ecological damage caused by less expensive open-pen systems is mostly unregulated (Lam 2016). A trend to incentivize ethical food systems, however, may be on the post-pandemic horizon.

## Strategic Conclusions

In this paper, we have assembled entry points for dialogue to re-imagine and re-structure food systems in post-pandemic times. We have presented foundational considerations of food ethics for strategic discussion on the future of the food system. This will require a transdisciplinary science in the empirical study and normative reflection of pro-social attitudes and value trade-offs as they affect diverse stakeholders and citizens along the global food value chain (from producer to consumer) (cf. Kaiser and Algers 2016; Lam 2019a). We have emphasized the fundamental importance of a deliberative framework that recognizes a plurality of values among food actors and facilitates open, respectful, and structured dialogue.

Thus, we unabashedly plea for a deliberative procedural ethics to radically transform current food systems. We stress the need to recognize the truly pervasive uncertainties and diversities in values and knowledge cultures around food, which makes a one-size-fits-all vision for future food systems (as promulgated in the EAT-*Lancet* report) an illusion. We argue for jump-starting open and transparent deliberations now, as underway in New Zealand, which mobilize diverse actors at local, regional and national scales. These deliberations should not be convened by political parties or governmental authorities, but rather, should prioritize non-partisan leadership. We also argue that the sciences need to move closer to a transdisciplinary mode of operation to be a truly constructive partner in these deliberations.

Our arguments embrace concepts of ethical soundness (Kaiser et al. 2007), deliberative democracy (Elstub 2010), good governance (van Doeveren 2011) and ethical governance (Lam et al. 2019). This notwithstanding, we outline what we call a deliberative framework, rather than align with a specific governance scheme, or even a normative ethical theory. We set our goal to sketch a general ethical framework that is understandable by readers from diverse disciplinary, cultural, and professional backgrounds. We focus attention on refining this ethical deliberative framework as a cornerstone in any future food governance system. The deliberation and decision-support tools introduced by Kaiser et al. (2007) and Lam et al. (2019) as part of an ethical governance scheme operationalize aspects of our framework. Further ethical decision-support and innovation tools based on bottom-up participatory schemes are being developed in, for example, open citizen (living) innovation labs (Leminen et al. 2012; Kuhlmann and Rip 2018). The development and implementation of such ethical tools may help realize our vision of structured ethical deliberation in science-for-policy.

COVID-19 has brought to the fore important value dimensions of our social realities (Lam in press), with a surprising degree of mutual understanding, solidarity and empathy in many societies and sectors therein. These are essential ingredients for social cohesion, which in turn is a prerequisite for ethical decision-making and behaviour. We hope and believe that the aftermath of the pandemic may provide a fruitful and productive ground for co-designing our food futures to be not only more sustainable, but also more ethical. We argue this can be achieved by bottom-up, regional, cross-sectoral and non-partisan deliberation to re-imagine and re-orient pre-pandemic food systems to be aligned with post-pandemic values and ethics.

## Footnotes

1 It is noteworthy that President-elect Joe Biden has announced that the USA will reclaim its place at the head of the table and be a coalition-builder under his leadership.

2 Globally, only four or five countries produce more rice than they eat; https://www.economist.com/briefing/2020/05/09/the-worlds-food-system-has-so-far-weathered-the-challenge-of-covid-19?fsrc=newsletter&utm_campaign=the-economist-today&utm_medium=newsletter&utm_source=salesforce-marketing-cloud&utm_term=2020-05-08&utm_content=article-link-1.

3 A post-normal science approach (Funtowicz and Ravetz 1990; Funtowicz and Ravetz 1993) promotes compromise, rather than consensus solutions, and consists of practical insights to assist scientists and knowledge users to work together. It includes an extended peer community that consists not only of diverse scientific experts, but also members of the public to represent all relevant stakeholder groups and concerned citizens. At its core, it is a participatory approach to science-for-policy that highlights inclusiveness in problem-framing, assessment, and quality control to limit potential biases in policy options and outcomes.

4 For instance, most of the certification systems for aquaculture are industry-driven, resulting in several hundreds of national and international schemes. Some NGOs, such as World Wide Fund for Nature and the Marine Stewardship Council, have produced sustainability certifications for seafood. Ethical considerations, however, are still rarely considered; but see Lam and Pitcher 2012, Lam 2016, Haugen et al. 2017.

5 Complex adaptive systems change and reorganize their component parts to adapt themselves to the problems posed by their surroundings (Holland 1992). An essential feature of such systems is nonlinearity, which leads to historical dependency and multiple possible dynamical outcomes (Levin 1998).

6 The Precautionary Principle is a controversial topic, subject to many interpretations and uses, but we adopt the UNESCO 2005 definition: “When human activities may lead to morally unacceptable harm that is scientifically plausible but uncertain, actions shall be taken to avoid or diminish that harm. Morally unacceptable harm refers to harm to humans or the environment that is threatening to human life or health, or serious and effectively irreversible, or inequitable to present or future generations, or imposed without adequate consideration of the human rights of those affected.” (UNESCO 2005, p.14). A version of this understanding is also the constitutional basis in the European Union since its Maastricht (1992) and Nice (2001) Treaties. Note that the dominant Anglo-American characterisations of the Precautionary Principle deviate from the European, and indeed UNESCO characterisation, in claiming that Precaution implies a no-risk strategy.

7 According to the FAO ( 2018a): “Agricultural production is limited by the increasing scarcity and diminishing quality of land and water resources, as well as by insufficient investment in sustainable agriculture. Climate change is increasingly affecting yields and rural livelihoods, while agriculture continues to emit greenhouse gases (GHGs).”

8 MK attended a pre-publishing review meeting of the SAPEA report on seafood ( 2017) that intended to include extended scientific data on fisheries and highlighted the limits of the official FAO data. The FAO representative broke out in a rage, exclaiming: “There is only one set of data, and that is the FAO data!”

9 Turner (Turner Jonathan 1997; p. 6) defines social institutions as “a complex of positions, roles, norms and values lodged in particular types of social structures and organising relatively stable patterns of human activity with respect to fundamental problems in producing life-sustaining resources, in reproducing individuals, and in sustaining viable societal structures within a given environment.”

10 The committee triangulated different assessments of diet-related disease mortality, each resulting in roughly 11 million lives saved. The statistics of diet-related mortality, however, operate with high uncertainties, especially when extended to a global level (see, e.g., Archer et al. 2017, Brown et al. 2014, and Ioannidis 2013).

11 Except for MEL, who wrote in lockdown from Norway.

12 See https://informedfutures.org/the-future-is-now/.

13 For elucidation of these points, see the *Koi Tū* document on the New Zealand food discussions (*Koi* Tū 2020).

14 Here, we adapt the quote from Antonio Gramsci: “The crisis consists precisely in the fact that the old is dying and the new cannot be born; in this interregnum a great variety of morbid symptoms appear.”

15 https://www.ft.com/content/f4fa76d9-aa11-4ced-8329-6fc8c250bc45

16 https://www.economist.com/briefing/2020/05/09/the-worlds-food-system-has-so-far-weathered-the-challenge-of-covid-19?fsrc=newsletter&utm_campaign=the-economist-today&utm_medium=newsletter&utm_source=salesforce-marketing-cloud&utm_term=2020-05-08&utm_content=article-link-1

17 A zoonosis is an infectious disease, such as COVID-19, caused by a pathogen that has jumped from a non-human animal (usually a vertebrate) to a human.

18 The average food loss per capita in Europe and the USA is between 280 and 300 kg/year, including loss at production. The per capita food waste per consumer in Europe is on average 95 kg/year (FAO 2018b).

19 An interesting European activity has emerged during the pandemic to reduce food waste: http://www.fao.org/save-food/regional/europeanunion/en/.
